# Mosquito net coverage in years between mass distributions: a case study of Tanzania, 2013

**DOI:** 10.1186/s12936-018-2247-z

**Published:** 2018-03-01

**Authors:** Zawadi M. Mboma, Hans J. Overgaard, Sarah Moore, John Bradley, Jason Moore, Dennis J. Massue, Karen Kramer, Jo Lines, Lena M. Lorenz

**Affiliations:** 1 Ifakara Health Institute, Bagamoyo, Tanzania; 2 Department of Disease Control, London School of Hygiene and Tropical Medicine, London, UK; 3 Faculty of Science and Technology, Norwegian University of Life Sciences, Ås, Norway; 4 Swiss Tropical and Public Health Institute, Basel, Switzerland; 5 University of Basel, Petersplatz 1, 4003 Basel, Switzerland; 6 MRC Tropical Epidemiology Group, London School of Hygiene and Tropical Medicine, London, UK; 7 National Institute for Medical Research, Muheza, Tanzania

**Keywords:** Long-lasting insecticidal nets (LLINs), Untreated nets, Universal coverage, Net ownership, Net access, Net use, Tanzania

## Abstract

**Background:**

The Government of Tanzania is the main source of long-lasting insecticidal nets (LLINs) for its population. Mosquito nets (treated and untreated) are also available in the commercial market. To sustain investments and health gains in the fight against malaria, it is important for the National Malaria Control Programme to monitor LLIN coverage especially in the years between mass distributions and to understand what households do if their free nets are deemed unusable. The aim of this paper was to assess standard LLIN indicators by wealth status in Tanzania in 2013, 2 years after the last mass campaign in 2011, and extend the analysis to untreated nets (UTNs) to investigate how households adapt when nets are not continuously distributed.

**Methods:**

Between October–December 2013, a household survey was conducted in 3398 households in eight districts in Tanzania. Using the Roll Back Malaria indicators, the study analysed: (1) household net ownership; (2) access to nets; (3) population net use and (4) net use:access ratio. Outcomes were calculated for LLINs and UTNs. Results were analysed by socio-economic quintiles and by district.

**Results:**

Only three of the eight districts had household LLIN ownership of more than 80%. In 2013, less than a quarter of the households had one LLIN for every two people and only half of the population had access to an LLIN. Only the wealthier quintiles increased their net ownership and access to levels above 80% through the addition of UTNs. Overall net use of the population was low (LLINs: 32.8%; UTNs: 9.5%) and net use:access ratio was below target level (LLINs: 0.66; UTN: 0.50). Both measures varied significantly by district.

**Conclusions:**

Two years after the last mass campaign, the percentage of households or population with access to LLINs was low. These findings indicate the average rate at which households in Tanzania lose their nets is higher than the rate at which they acquire new nets. The wealthiest households topped up their household net ownership with UTNs. Efforts to make LLINs available through commercial markets should be promoted, so those who can afford to buy nets purchase LLINs rather than UTNs. Net use was low around 40% and mostly explained by lack of access to nets. However, the use:access ratio was poor in Mbozi and Kahama districts warranting further investigations to understand other barriers to net use.

**Electronic supplementary material:**

The online version of this article (10.1186/s12936-018-2247-z) contains supplementary material, which is available to authorized users.

## Background

Since the global resurgence of interest in malaria control about 20 years ago, insecticide-treated nets (ITNs) have been the most widely distributed intervention against malaria and account for a 68% decline in *Plasmodium falciparum* infection prevalence in sub-Saharan Africa [1]. Universal coverage as recommended by the World Health Organization (WHO) is defined as “universal access to, and use of, long-lasting insecticidal nets (LLINs)” of all people at risk of malaria, and is defined operationally as one net for every two people [2]. Tanzania has a long-standing record in the deployment of mosquito nets as an intervention for malaria control [3–7]. The use of ITNs in Tanzania has been associated with the reduction of malaria morbidity and mortality, particularly in children under the age of five [8, 9].

Mass distribution campaigns are the primary source of LLINs in most malaria endemic countries and aim to ensure equitable distribution across all socio-economic groups [1, 10–12]. Given the increasing distribution of large numbers of mosquito nets in communities, the Roll Back Malaria Monitoring and Evaluation Reference Group (MERG) developed indicators to assess and compare LLIN interventions in countries at risk of malaria [13]. Household surveys are widely used to measure the MERG indicators, which determine achievements of universal coverage of LLINs following mass distributions [13].

Between 2004 and 2014, the Government of Tanzania distributed nets to pregnant women and infants at a subsidised cost during their routine antenatal and immunization clinic visits through the Tanzania National Voucher Scheme (TNVS) [14–16]. Nationwide, children under the age of 5 received nets free of charge through the Under-Five Catch-up Campaign (U5CC) between 2009 and 2010 [17], and a Universal Coverage Campaign (UCC) was implemented in 2010 and 2011 to reach all remaining uncovered sleeping spaces [18]. Another mass universal replacement campaign (URC) was conducted between 2015 and 2017 to achieve universal coverage in most of the country. Since 2013, the School Net Programme (SNP) has been ongoing in the Southern Zone to explore sustainable continuous “Keep Up” mechanisms to distribute nets into the community [19, 20, 21].

In addition, both insecticidal and untreated mosquito nets (UTNs) are available through the private sector at varying costs [22]. A to Z Textile Mills Ltd. holds the biggest market share for mosquito nets in Tanzania, but their commercial market is currently restricted to UTNs (Safinet) and supplies to international funders for mass LLIN campaigns (Olyset and Miranet) within the region and elsewhere (Nick Brown, Business Development Manager, *pers. comm.*). There are three more local manufacturers of UTNs than LLINs in Tanzania, which increases the accessibility and availability of UTNs in the commercial markets at a cheaper cost [22]. Though not as efficient as LLINs for protection against malaria, UTNs do provide physical protection against mosquitoes if in relatively good condition [8, 23–25].

While many studies focus on evaluating the achievements of the LLIN distributions usually immediately following mass campaigns [12, 26–30], this study provides, (1) data on LLIN coverage at a unique time between mass campaigns, and (2) an account of how households adapt when nets are not freely distributed, including the acquisition of UTNs. Using the MERG indicators, LLIN and UTN ownership, access and use was assessed to investigate the net landscape of Tanzania 2 years since the last mass campaign with particular emphasis on how the population responds to loss of free LLINs and whether this is affected by socio-economic status. The National Malaria Control Programme (NMCP) could use these data to predict current LLIN coverage following the URC in 2015–2017 to better assess target areas and populations for continuous net distribution strategies.

## Methods

### Study sites and population sampling

**Fig. 1:**
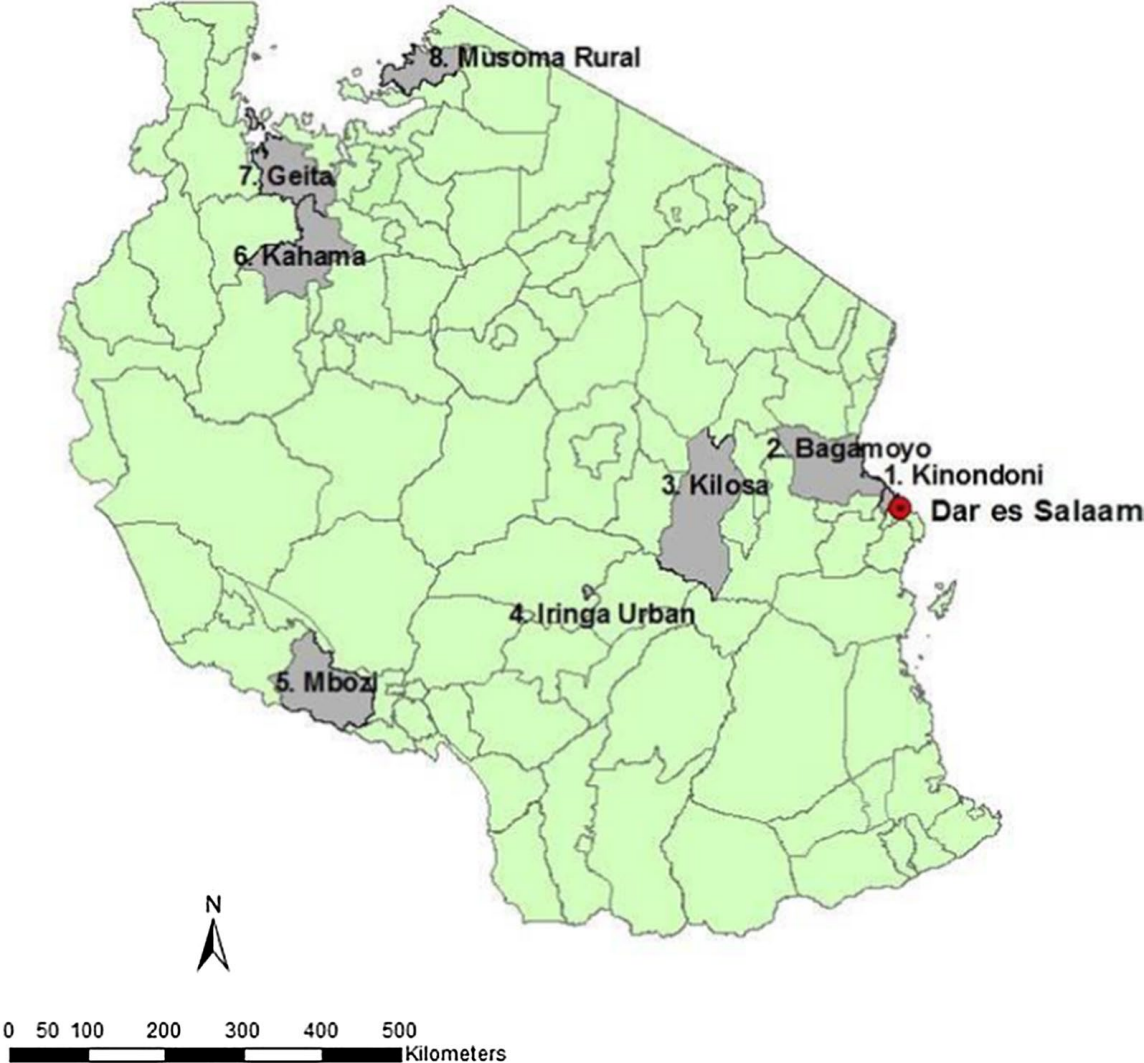
Geographical distribution of the eight districts in Tanzania sampled for this study. The eight districts sampled in this study were: (1) Kinondoni, (2) Bagamoyo, (3) Kilosa, (4) Iringa Urban, (5) Mbozi, (6) Kahama, (7) Geita, and (8) Musoma Rural

The study was conducted in eight districts in Tanzania (Fig. 1) between October and December 2013, during the baseline survey of a long-term LLIN durability study [31]. The eight districts were selected from 23 districts enrolled in the Sentinel Panel of Districts (SPD) for the Sample Vital registration with Verbal Autopsy (SAVVY) project [32], a demographic surveillance platform based at the Ifakara Health Institute (IHI). The eight districts were selected to represent six of the eight geographical zones of Tanzania with varying malaria prevalence across study sites, excluding the Southern Zone (ongoing SNP) and the Northern Zone (low malaria prevalence at the time) [33]. This study was conducted leading into the short rainy season when transmission is usually lowest. Of the eight districts, two (Kinondoni and Iringa) were urban while the other six were rural. Ten villages in each district were selected for inclusion except for Kinondoni district where only six villages were available. In each selected village, 45 households were randomly selected from the SAVVY database, giving a total of 3420 households. The sample size calculation was for the overall long-term LLIN durability study outcomes [31].

### Data collection

A cross-sectional household survey was conducted. The household questionnaire was programmed using Open Data Kit (ODK) [34] and administered using Google Nexus tablet computers. The questionnaire included a household member roster and questions about the mosquito net(s) owned and whether the net(s) had been used the previous night. The number of sleepers under each net the previous night was recorded. Each mosquito net identified in the household was assigned a unique barcode. All participating households were provided with new LLINs to cover all sleeping spaces as part of their enrolment into the net durability study [31]. All mosquito nets present in these households were collected and returned to the IHI laboratories in Bagamoyo where they were sorted by colour, size, product label and manufacturing date (creating a “net database”). The insecticide treatment status of each net was identified using its attached product label and categorized as either LLIN, UTN or unknown (if label was missing). The net database was linked to the questionnaire data using the unique barcode assigned to each mosquito net collected.

### Data analysis

#### Mosquito net indicators

This study used the MERG indicators to report the status of Tanzania’s mosquito net coverage in 2013 (Table 1) [13]. Household net ownership, which is defined as the percentage of households owning at least one net, one LLIN or UTN, was determined. The percentage of households with at least one net for every two people in its household (“households with enough nets”) was also determined for LLINs, any net and UTNs. “Population access”, i.e. the percentage of the population with potential to be protected by a net within their household, assuming a net can be used by two people was determined for LLINs, any net and UTNs (values were corrected to a maximum value = 1 to ensure the value for potential users does not exceed the number of actual household members [A. Kilian pers. comm.]). Population access was calculated using the following equation:

$$Population \;Access = \frac{{{\text{Number of nets present in household}}\; *\;2}}{\text{Number of people who slept in the household the previous night}}$$

**Table 1:** Descriptions of mosquito net indicators used

Mosquito net indicator	Indicator description
Household ownership	Percentage of households owning at least one net, one LLIN, or one untreated net
Household with enough nets	Percentage of households with at least one net, one LLIN, or one untreated net, for every two people
Population access	Percentage of the population with access to any net, LLIN, or untreated net within their household, assuming each net is used by two people
Population net use	Percentage of the population that used any net, any LLIN, or any untreated net the previous night
Net use:access ratio	Percentage of the population that used a net the previous night divided by the percentage of the population that had access to a net
Net use gap	The proportion of the population who had access to a net within their household, assuming each net is used by two people, but did not sleep under one (1-use:access ratio)

The proportion of the population that reported to have used a net, an LLIN or UTN, the previous night was calculated.

The use:access ratio was calculated by dividing the percentage of the population that reportedly used a net the previous night by the percentage of the population that had access to a net. The mean number of sleepers per net was calculated by multiplying the use:access ratio by two, assuming each net should be used by two people. The net use gap (“1-use:access ratio” [28]), i.e. the proportion of the population who had access to a net within their household, assuming each net is used by two people, but did not sleep under one, was also determined. The net use gap indicates whether people made a choice not to sleep under a net despite having access or whether they were without access to nets in their households [28].

#### Socio-economic status

The socio-economic status (SES) of each participating household was calculated by creating a wealth index based on measures such as the materials used to construct the house, household amenities and assets owned [35]. Questions to measure assets were adapted from the WHO sample questionnaire for monitoring LLIN durability under operational conditions [36] to fit the current local context. Using principal component analysis (PCA) [37], a weighted score was calculated for each household and the whole population divided into five quintiles, following the methods described by the Demographic Health Survey Comparative Report No. 6 [38].

#### Statistical analysis

Data analysis was carried out using statistical software package STATA 13.1 (StataCorp LP, College Station, TX). Using the survey suite of commands to account for the clustered sampling design, a single-stage sampling scheme designated the variable ‘village’ as the primary sampling unit. This was done to account for the highest level of clustering (village) to give the correct standard errors even if the lower levels of clustering (household) were not explicitly modelled [39]. Statistical analysis focused on the effect of socio-economic status on the variation between access to and use of any net (treated and untreated) and LLINs. Logistic regressions were performed to analyse the effect of SES on the following dependent variables: (1) ownership of at least one net (any type), (2) ownership of at least one LLIN, (3) ownership of at least one UTN, (4) households with enough nets (any type), (5) households with enough LLINs, (6) households with enough UTNs, (7) population access to any net within the household, (8) population access to an LLIN within the household, (9) population access to an UTN within the household, (10) population net use the previous night, (11) population LLIN use the previous night, (12) population use of UTNs the previous night, (13) any net use:access ratio, (14) LLIN use:access ratio, and (15) UTN use:access ratio, adjusting for district variation (Table 2).

**Table 2:** Number (%) of households by socio-economic quintiles (SES) in the eight districts in Tanzania, 2013

District	Socio-economic quintiles (SES)					Total
	Poorest	Second poorest	Medium	Wealthier	Wealthiest	
Bagamoyo (R)	66 (15.0)	77 (17.5)	114 (26.0)	126 (28.8)	55 (12.6)	438 (100)
Kinondoni (U)	0 (0.0)	0 (0.0)	2 (0.7)	25 (9.3)	242 (90.0)	269 (100)
Kilosa (R)	124 (27.6)	80 (17.8)	85 (18.9)	118 (26.3)	42 (9.4)	449 (100)
Iringa (U)	0 (0.0)	4 (0.9)	24 (5.4)	144 (32.1)	277 (61.7)	449 (100)
Mbozi (R)	49 (10.9)	125 (27.8)	162 (36.1)	95 (21.2)	18 (4.0)	449 (100)
Kahama (R)	164 (36.6)	113 (25.2)	70 (15.6)	64 (14.3)	37 (8.3)	448 (100)
Geita (R)	131 (29.2)	131 (29.2)	120 (26.7)	65 (14.5)	2 (0.5)	449 (100)
Musoma (R)	146 (32.7)	150 (33.6)	102 (22.8)	43 (9.6)	6 (1.3)	447 (100)
Total	680 (20.0)	680 (20.0)	679 (20.0)	680 (20.0)	679 (20.0)	3398 (100)

*R* rural, *U* urban

Variations between net use and access among different districts was assessed for LLINs only. This is because the WHO specifically recommends universal coverage with LLINs [2].

## Results

**Fig. 2:**
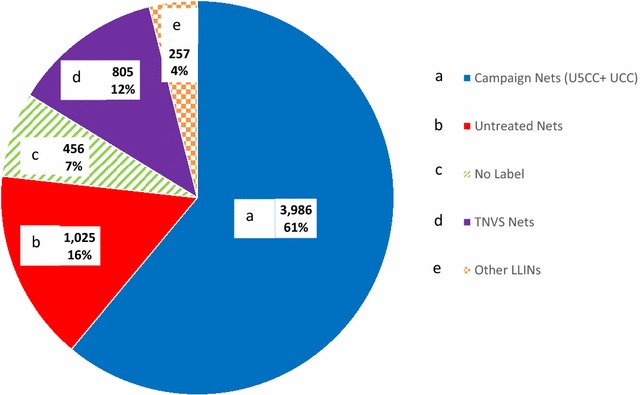
Assessment of 6529 nets collected from households. **a** Campaign Nets: Under-Five Catch-Up Campaign (U5CC) and Universal Coverage Campaign (UCC); **b** untreated nets; **c** no label; **d** Tanzania National Voucher Scheme (TNVS); and **e** other LLINs

A total of 6529 nets were collected from 3398 households from 76 villages across eight districts in Tanzania [40]. Seventy-seven percent of nets were LLINs, 16% UTNs, and 7% had no labels attached (Fig. 2). The predominant net product was Olyset (74.2%). Other LLIN products included PermaNet (1.5%) and BASF (0.9%). Untreated net products included Safinet (13.5%), SupaNet (1.5%) and Health Net Ltd (0.5%). Seventy-three percent of all nets collected were identified by their colour to have come from a government distribution mechanism (TNVS, U5CC or UCC) (Fig. 2). Of the 3986 campaign nets identified, only 1063 could be distinguished by manufacturing date (U5CC: 135, UCC: 928), the rest had lost their manufacturing label. Of the 6529 nets collected, 85% were single size (3 × 6 feet) while 15% were double size (4 × 6 feet) in dimensions. Eighty-five percent of the single size nets were LLINs. Fifty-one percent of the double-sized nets were UTNs, 35% were LLINs and 14% unknown. Ninety-seven percent of nets were square in shape while 3.3% were conical-shaped. Seventy-one percent of the conical-shaped nets were UTNs.

Most of the households in Kinondoni and Iringa (urban districts) ranked among the wealthiest SES quintile while none ranked among the poorest quintile (Table 2). Household ownership of at least one government-distributed LLIN (TNVS, U5CC or UCC) was almost twice as high among the poorest quintile at 90.0% [95% CI 86.2–92.8%] compared to the wealthiest quintile at 47.3% [95% CI 42.1–52.6%]. Thirty-five percent of households owned both an LLIN and a UTN.

### Net ownership

**Fig. 3:**
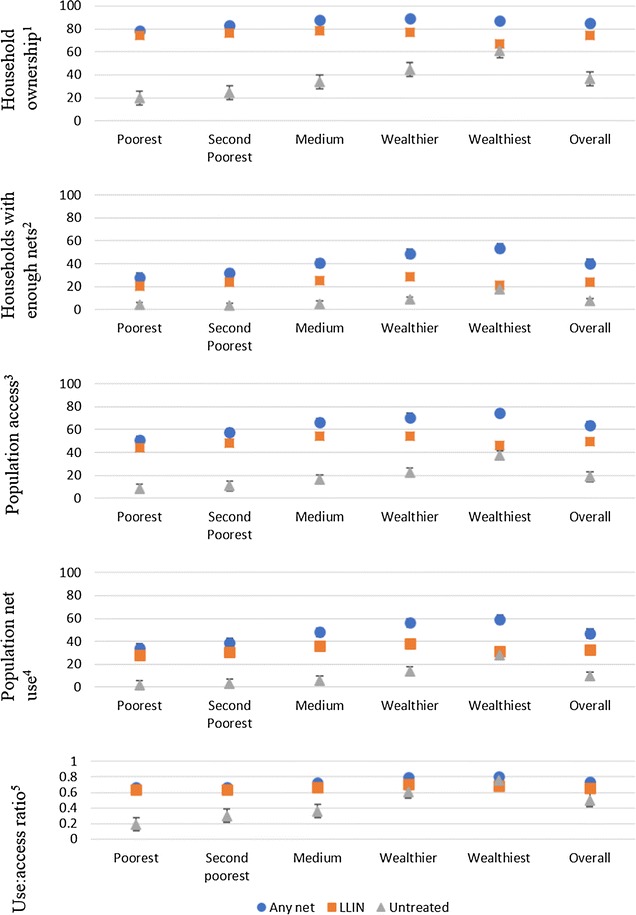
Ownership, access, and use of any nets, LLINs and UTNs by socio-economic quintile. The mean percentage household ownership, access and use of any nets, LLINs and UTNs by socio-economic quintile in Tanzania, October–December 2013 (also see Additional file 1 for tabulated data). Error bars represent 95% confidence intervals. Definitions of mosquito net indicators used are listed in Table 1

Overall, 85.0% [95% CI 82.3–87.4%] of households owned at least one net (any type) while 74.5% [95% CI 71.0–77.7%] and 36.7% [95% CI 32.6–41.0%] of households owned at least one LLIN and at least one UTN, respectively (Fig. 3). The wealthiest quintiles had the highest percentage of household net ownership at 89.3% [95% CI 85.3–92.3%] but the lowest percentage of households owning at least one LLIN at 66.6% [95% CI 59.2–73.2%] (Fig. 3). The poorest quintile had the lowest household ownership of any net at 78.1% [95% CI 70.8–84.0%] while the middle quintile had the highest LLIN ownership at 78.6% [95% CI 72.8–83.5%] (Fig. 3). Ownership of UTNs increased with the increase of wealth quintile.

Socio-economic status was significantly positively associated with ownership of any net (Table 3). For those in the wealthiest quintile, the odds of owning a net was 2.62 times the odds of owning any net for those in the lowest quintile. There was no statistically significant association between SES and LLIN ownership. However, the odds of the middle quintile to own an LLIN was 1.47 times the odds of owning an LLIN for those in the lowest quintile. Socio-economic status was significantly positively associated with ownership of UTNs. The odds of the wealthiest quintile to own a UTN was 6 times the odds of owning an UTN for those in the lowest quintile (Table 3).

**Table 3:** The effect of SES on mosquito net indicators for any net, LLINs and untreated nets

Mosquito net indicator	Variable	SES	Unadjusted odds ratio (95% CI)	P value	Adjusted odds ratio^a^ (95% CI)	P value
Household ownership	Any net	Poorest	1	0.013	1	0.005
		Second Poorest	1.38		1.53	
		Medium	2.01		2.33	
		Wealthier	2.33		2.61	
		Wealthiest	1.86		2.62	
	LLIN	Poorest	1	0.070	1	0.053
		Second Poorest	1.13		1.25	
		Medium	1.29		1.47	
		Wealthier	1.15		1.26	
		Wealthiest	0.7		0.87	
	Untreated net	Poorest	1	0.000	1	0.000
		Second poorest	1.31		1.36	
		Medium	2.08		2.18	
		Wealthier	3.24		3.35	
		Wealthiest	6.19		6.95	
Household with enough nets	Any net	Poorest	1	0.000	1	0.001
		Second poorest	1.2		1.22	
		Medium	1.76		1.67	
		Wealthier	2.46		2.04	
		Wealthiest	2.97		2.47	
	LLIN	Poorest	1	0.039	1	0.121
		Second poorest	1.18		1.21	
		Medium	1.27		1.2	
		Wealthier	1.53		1.29	
		Wealthiest	1.04		0.92	
	Untreated net	Poorest	1	0.000	1	0.002
		Second poorest	0.88		0.81	
		Medium	1.31		1.10	
		Wealthier	2.30		1.78	
		Wealthiest	5.09		3.41	
Population access	Any net	Poorest	1	0.005	1	0.005
		Second poorest	1.40		1.53	
		Medium	2.06		2.31	
		Wealthier	2.58		2.68	
		Wealthiest	1.92		2.43	
	LLIN	Poorest	1	0.039	1	0.021
		Second poorest	1.15		1.24	
		Medium	1.31		1.44	
		Wealthier	1.26		1.25	
		Wealthiest	0.7		0.77	
	Untreated net	Poorest	1	0.000	1	0.000
		Second poorest	1.33		1.35	
		Medium	2.15		2.17	
		Wealthier	3.51		3.40	
		Wealthiest	6.52		6.68	
Population net use	Any net	Poorest	1	0.000	1	0.000
		Second poorest	1.23		1.3	
		Medium	1.8		1.93	
		Wealthier	2.49		2.52	
		Wealthiest	2.82		2.92	
	LLIN	Poorest	1	0.009	1	0.002
		Second poorest	1.13		1.19	
		Medium	1.44		1.51	
		Wealthier	1.54		1.56	
		Wealthiest	1.18		1.23	
	Untreated net	Poorest	1	0.000	1	0.000
		Second poorest	2.05		2.25	
		Medium	3.82		4.08	
		Wealthier	9.62		8.17	
		Wealthiest	23.47		18.89	
Use:access ratio	Any net	Poorest	1	0.014	1	0.050
		Second poorest	1.04		1.23	
		Medium	1.13		1.28	
		Wealthier	1.62		1.77	
		Wealthiest	1.85		1.7	
	LLIN	Poorest	1	0.771	1	0.899
		Second poorest	0.83		0.94	
		Medium	0.88		0.99	
		Wealthier	1.01		1.11	
		Wealthiest	1.07		0.97	
	Untreated net	Poorest	1	0.721	1	0.321
		Second poorest	1.31		3.47	
		Medium	1.97		1.25	
		Wealthier	2.02		0.69	
		Wealthiest	2.37		0.76	

See Table 1 for definitions of mosquito net indicators

^a^Adjusted for district

### Households with one net for every two people

Overall, the percentage of households with enough LLINs to cover every two of its household members was low (Fig. 3). Only in the wealthiest quintile did more than half of the households have enough nets (any type) for everyone in the household at 53.3% [95% CI 48.7–57.9%]. The percentage of households with at least one LLIN for every two people was below 30% across all socio-economic quintiles. The odds of the wealthiest quintile to have households with enough nets of any type was 2.47 times the odds for those in the lowest quintile, but there was no statistically significant effect of SES on household access to LLINs (Table 3). There was a significantly positive association between SES and households with enough UTNs (Table 3).

### Population access

The wealthier quintiles had the highest percentage of their population with access to a net (any net: 74.3% [95% CI 69.2–79.4%]; LLINs: 54.3% [95% CI 49.5–59.0%]; UTNs: 60.5% [95% CI 55.4–65.9%] (Fig. 3)). Socio-economic status was significantly associated with population access to all nets (treated and untreated) (Table 3). For LLINs, the middle quintile had the highest odds of its populations having access while the wealthiest had the lowest odds.

### Population net use

The average number of people sleeping under any net was 1.8 with 43.1% of nets having only 1 sleeper while 54.5% with 2–3 sleepers under one net. The average number of people sleeping under an LLIN was 1.8 with 31.4% of the LLINs having only one sleeper while 39.4% of LLINs had 2–3 sleepers. The mean number of sleepers under UTNs was 1.7 with 34.5% having only one sleeper under it while 38.1% had 2–3 sleepers.

Population net use was lowest in the poorest quintile regardless of the net’s insecticide-treatment status (any net: 33.9% [95% CI 27.9–39.8%]; LLIN: 28.2% [95% CI 23.2–33.2%]; UTNs: 1.6% [95% CI 0.6–2.6%] (Fig. 3)). Socio-economic quintile was significantly associated with population net use. The odds of the wealthiest households compared to the odds of the poorest households using nets was 3 times for any net, 1.2 times for an LLIN and 18.89 times for a UTN (Table 3).

### Use:access ratio and net use gap

The overall proportion of people that had access to a net and slept under it the previous night was 0.73 for any net, 0.66 for LLINs, and 0.50 for UTNs (Fig. 3). The net use gap ranged between 0.20–0.33 for any net, 0.30–0.36 for LLINs, and 0.25–0.81 for UTNs depending on the socio-economic quintile (Fig. 3). The odds of the wealthiest individuals to sleep under any net if they had access to it was 1.7 times the odds of sleeping under any net for the poorer individuals. There was no statistically significant association between socio-economic status and LLIN use:access ratio (Table 3).

### District variation of LLIN coverage

**Fig. 4:**
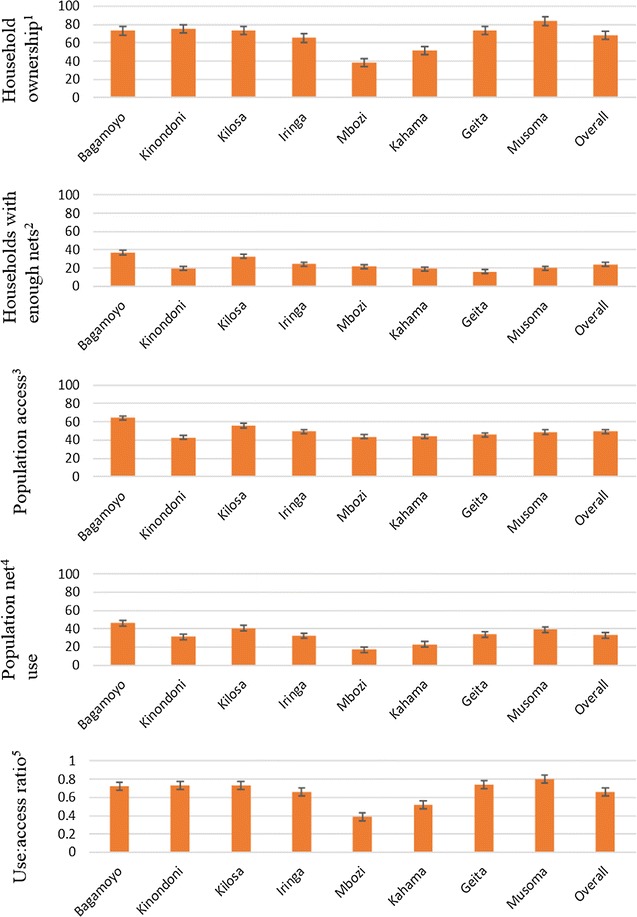
Ownership, access, and use of LLINs by district in Tanzania, October–December 2013. The mean percentage household ownership, access and use of LLINs by district in Tanzania, October–December 2013 (also see Additional file 2 for tabulated data). Error bars represent 95% confidence intervals. Definitions of mosquito net indicators used are listed in Table 1

Overall, households with enough LLINs for every two of its household members were 23.8% [95% CI 21.2–26.7%], the percentage of the population with access to an LLIN within their household was 49.2% [95% CI 46.3–52.0%], and the percentage of the population that used an LLIN the previous night was 38.2% [95% CI 29.9–35.8%] (Fig. 4). The overall use:access ratio of LLINs was 0.66 and in turn the LLIN use gap was 0.34.

Only three districts, namely Bagamoyo, Kilosa and Musoma had more than 80% of households owning at least one LLIN (Fig. 4). Kinondoni district had the lowest percent of household ownership of LLINs at 62.5% [95% CI 40.5–80.3%] while neighbouring Bagamoyo had the highest at 83.3% [95% CI 74.3–89.6%]. Geita had the lowest percentage of households with enough LLINs at 16.0% [95% CI 12.2–20.8%] and low population access at 45.6% [95% CI 40.7–50.5%]. Mbozi and Kahama districts, who have the lowest household ownership of LLINs, had the lowest LLIN use:access ratios of 0.39 and 0.52 respectively while Musoma district had the highest at 0.80 (Fig. 4).

## Discussion

Overall, the percentage of households with one LLIN for every two people was below 30%. This finding indicates that 2 years after the mass distribution, many households were without enough nets to cover their population, leading to low population access to LLINs (below 50%). This emphasizes that the URC was long overdue by 2013. Recent national surveys suggest that malaria prevalence in Tanzania may have increased from 9.2% in 2011–2012 to 14.4% in 2015–2016 [33, 41], which could be attributed to poor LLIN indicators although the difference in malaria prevalence could also be attributed to varying transmission intensity between the survey years [42, 43]. The WHO currently recommends mass distribution campaigns to be conducted at 3-year intervals unless there is reliable data to justify longer replacement intervals or as per locally available investments to accommodate population growth and intermittent net loss [2]. This study emphasizes the need for continuous malaria intervention especially during the gap years between mass distributions. Geita district, for example, recorded the highest malaria prevalence (38.4%) in 2015–2016 [41] and lowest percentage of households with enough LLINs (16%) in this study. It is currently profiting from the expansion of SNP to the Western and Lake Zone since late 2016 to maintain high net coverage [44].

Generally, household ownership of any type of net was highest among the wealthiest quintile (89.3%). Sixty percent of the wealthiest households owned at least one UTN, most probably acquired from the commercial market. This indicates willingness to purchase affordable nets for continued protection against mosquitoes in the absence of free net distributions. A literature review by Koenker and Yukich [45] found that households tend to use the nets available to them irrespective of net characteristics (colour, shape, size or texture), probably because they are restricted to what is distributed or what they have access to. Purchasing their own nets, however, allowed households to exercise choice regarding treatment status, material and size of net. This assessment found that 51% of the double-size nets and 71% of the conical-shaped nets were UTNs.

The inequalities observed across socio-economic quintiles in the acquisition of UTNs was similar to what was observed in Nigeria [46]. The wealthiest households, situated in the urban districts of Kinondoni and Iringa, increased their household access to nets through the commercial markets. Access to a variety of products and affordable prices have been shown to have a significant association with willingness to purchase mosquito nets in Ethiopia [47]. Remotely-located districts are often disadvantaged by increased costs to cover transport charges [16]. This study found that household ownership of at least one government-distributed LLIN (TNVS, U5CC, UCC), distributed 2–4 years prior to this study, was almost twice as high in the poorest quintile (90%) compared to the wealthiest quintile (47%). This indicates that households belonging to the lower socio-economic quintiles relied mostly on campaign LLINs and kept them for longer. Hence, there is a need to identify pro-poor methods of targeting net distributions such as the SNP to lower socio-economic quintiles to ensure households have enough nets to cover all members.

It will be important to identify locally and culturally appropriate avenues for behavioural-change campaigns (BCC) to motivate increased purchasing of LLINs while strengthening the local production of LLINs through private–public partnerships [22, 48, 49]. It is also useful to explore factors associated with net retention and how those can be incorporated in the BCC in districts with high net loss. Household net ownership of at least one LLIN in Mbozi district dropped by 28.8% from what was reported by the THMIS 2011–2012, 10 months prior to this study [33].

Population net use of any net type and LLINs was low across all socio-economic quintiles. Any net use was highest among the wealthiest quintile but was still below 60%. Overall, LLIN use:access ratio of 0.66 indicated that not all of the nets collected from households were used [29]. Previous studies have identified reasons for net non-use include lack of access to nets [50, 51] or discomfort, low mosquito density, or sleeping elsewhere [52, 53]. Across districts, the LLIN use:access ratio was lowest in Mbozi at 0.39 (mean number of people per net was 0.7). Mbozi district is in the Southern Highlands, a hypo-endemic zone (with less than 3 months of transmission a year, < 10% malaria prevalence in children 2–9 years old) [54, 55]. Thus, people might not see malaria as a public health threat, explaining the low use rate. Further studies need to be conducted to understand the barriers to net use in specific geographical areas, especially following the informative “Hang Up” campaign by the Tanzania Red Cross Society after the UCC [56].

This study was unable to match net use with user characteristics such as age and gender from the household member roster. Therefore, it was not possible to analyse the person-type most and least likely to sleep underneath a net, to understand those most likely to remain uncovered that ought to be targeted in future net distributions [57–59]. The uneven distribution of SES quintiles observed after PCA analysis where most of the households in Kinondoni and Iringa (urban districts) ranked among the wealthiest while no household ranked among the poorest (Table 2), is an important limitation of this study. However, statistical analysis controlled for the variation observed between districts. Decision-makers should adjust by district SES-focused interventions and consult with the Tanzania Social Action Fund on the modalities of pro-poor focused interventions [60].

## Conclusions

In 2013, 2 years after the last mass campaign and 2 years before the URC, the percentage of households or populations with access to LLINs, assuming each LLIN is used by two people, was low (< 30 and < 50%, respectively). These findings indicate that the average rate at which households in Tanzania lose their nets is higher than the rate at which they acquire new nets. There is a need for continuous distribution of LLINs, especially during gap years between mass distributions. The NMCP is currently implementing continuous “Keep Up” strategies delivering LLINs free of charge through the expanding SNP, and through routine health care to pregnant women at their first antenatal clinic (ANC) and at an infant’s first vaccination clinic. Household ownership of any type of net was highest among the wealthier quintile (89.3%), who topped up their ownership with UTNs. Efforts to make LLINs available through commercial markets should be promoted, so that those who can buy nets from the market purchase LLINs rather than UTNs. Targeted BCC is crucial to motivate net use among those with access to nets within their households. Further investigation is recommended to understand barriers to net use and what can be done to ensure year-round net use.

## Additional files

**Additional file 1.** Tabulated data representing household ownership, access and use of any nets, LLINs and UTNs by socio-economic quintile in Tanzania, October–December 2013 also presented in Fig. 3. Definitions of mosquito net indicators are listed in Table 1.

**Additional file 2.** Tabulated data representing household ownership, access and use of LLINs by district in Tanzania, October–December 2013 also presented in Fig. 4. Definitions of mosquito net indicators are listed in Table 1.

## Abbreviations

IHI: Ifakara Health Institute
ITN: insecticide-treated net
LLIN: Long-lasting insecticidal net
MERG: Roll Back Malaria Monitoring and Evaluation Group
NMCP: National Malaria Control Programme
ODK: Open Data Kit
PCA: principal component analysis
SAVVY: Sample Vital registration with Verbal Autopsy
SES: Socio-economic status
SNP: School-Net Programme
SPD: Sentinel Panel of Districts
THMIS: Tanzania HIV/AIDS and Malaria Indicator Survey
TNVS: Tanzania National Voucher Scheme
U5CC: Under-Five Catch-up Campaign
UCC: Universal Coverage Campaign
URC: universal replacement campaign
UTN: untreated net
WHO: World Health Organization
