# Supplementary material for: Mosquito net coverage in years between mass distributions: a case study of Tanzania, 2013
Source: Malar J. 2018 Mar 1;17:100. doi: 10.1186/s12936-018-2247-z (PMC5831856; doi:10.1186/s12936-018-2247-z)
Supplement: Supplementary file 1 — Additional file 1. Tabulated data representing household ownership, access and use of any nets, LLINs and UTNs by socio-economic quintile in Tanzania, October–December 2013 also presented in Fig. 3. Definitions of mosquito net indicators are listed in Table 1. [file 12936_2018_2247_MOESM1_ESM.pdf]

Table 1: Ownership, access, and use of nets (any type) by socio-economic quintile in Tanzania, October – December, 2013

Socio-economic quintile	Household ownership[1]	Households with enough nets[2]	Population access[3]	Population net use[4]	Use:access ratio[5]
Poorest	78.1 (70.8-84.0)	27.8 (22.5-33.8)	50.8 (44.7-56.9)	33.9 (27.9-39.8)	0.67
Second Poorest	83.1 (79.5-86.1)	31.6 (27.4-36.1)	57.7 (54.0-61.4)	38.6 (33.5-43.7)	0.67
Medium	87.8 (84.2-90.7)	40.4 (35.9-45.0)	66.3 (62.6-70.0)	47.9 (42.3-53.6)	0.72
Wealthier	89.3 (85.3-92.3)	48.7 (42.1-55.3)	70.7 (66.0-75.4)	56.0 (50.2-61.8)	0.79
Wealthiest	86.9 (82.0-90.6)	53.3 (48.7-57.9)	74.3 (69.2-79.4)	59.1 (53.6-64.5)	0.80
**Overall**	**85.0 (82.3-87.4)**	**40.3 (36.5-44.3)**	**63.8 (60.2-67.3)**	**43.5 (39.2-47.8)**	**0.73**

[1-5] Descriptions of mosquito net indicators are listed on Table 1

Table 2: Ownership, access, and use of LLINs by socio-economic quintile in Tanzania, October – December, 2013

Socio-economic quintile	Household ownership[1]	Households with enough nets[2]	Population access[3]	Population net use[4]	Use:access ratio[5]
Poorest	74.1 (67.0-80.2)	20.7 (16.2-26.2)	44.0 (38.8-49.2)	28.2 (23.2-33.2)	0.64
Second Poorest	76.5 (71.8-80.6)	23.5 (19.9-27.6)	48.4 (44.8-52.0)	30.8 (26.7-35.0)	0.64
Medium	78.6 (72.8-83.5)	24.9 (21.3-28.9)	54.0 (49.9-58.1)	36.1 (31.3-41.0)	0.67
Wealthier	76.8 (71.7-81.1)	28.5 (23.9-33.6)	54.3 (49.5-59.0)	37.8 (33.3-42.4)	0.70
Wealthiest	66.6	21.4	45.8	31.6	0.69

	(59.2-73.2)	(17.2-26.2)	(40.7-51.0)	(28.2-35.0)	
**Overall**	**74.5** **(71.0-77.7)**	**23.8** **(21.2-26.7)**	**49.2** **(46.3-52.0)**	**32.8** **(29.9-35.8)**	**0.66**

[1-5] Descriptions of mosquito net indicators are listed on Table 1

Table 3: Ownership, access, and use of untreated nets by socio-economic quintile in Tanzania, October – December, 2013

Socio-economic quintile	Household ownership[1]	Households with enough nets[2]	Population access[3]	Population net use[4]	Use:access ratio[5]
Poorest	19.9 (16.1-24.2)	4.0 (2.6-6.0)	8.3 (6.3-10.2)	1.6 (0.6-2.6)	0.19
Second Poorest	24.6 (20.4-29.3)	3.5 (2.0-6.1)	10.9 (8.8-13.0)	3.3 (1.7-4.9)	0.30
Medium	34.0 (28.9-39.5)	5.2 (3.1-8.4)	16.4 (13.3-19.5)	5.9 (3.4-8.5)	0.36
Wealthier	44.6 (39.7-49.5)	8.7 (6.4-11.5)	22.6 (19.9-25.4)	13.7 (9.7-17.7)	0.61
Wealthiest	60.5 (55.4-65.4)	17.4 (14.1-21.2)	37.4 (33.9-40.9)	27.9 (23.1-32.8)	0.75
**Overall**	**36.7** **(32.6-41.0)**	**7.7** **(6.1-9.7)**	**18.9** **(16.0-21.9)**	**9.5** **(6.8-12.2)**	**0.50**

[1-5] Descriptions of mosquito net indicators are listed on Table 1
