# Supplementary material for: Mosquito net coverage in years between mass distributions: a case study of Tanzania, 2013
Source: Malar J. 2018 Mar 1;17:100. doi: 10.1186/s12936-018-2247-z (PMC5831856; doi:10.1186/s12936-018-2247-z)
Supplement: Supplementary file 2 — Additional file 2. Tabulated data representing household ownership, access and use of LLINs by district in Tanzania, October–December 2013 also presented in Fig. 4. Definitions of mosquito net indicators are listed in Table 1. [file 12936_2018_2247_MOESM2_ESM.pdf]

Table 1: Ownership, access and use of LLINs by district in Tanzania, October - December, 2013

District	Household ownership[1]	Households with enough nets[2]	Population access[3]	Population net use[4]	Use:access ratio[5]
Bagamoyo	83.3 (74.3-89.6)	36.8 (28.7-45.6)	64.3 (56.3-72.3)	46.0 (38.4-53.6)	0.72
Kinondoni	62.5 (40.5-80.3)	19.3 (12.5-28.6)	42.6 (27.8-57.4)	31.1 (22.2-39.9)	0.73
Kilosa	81.3 (72.5-87.7)	32.5 (23.7-42.7)	55.7 (45.6-65.9)	40.5 (29.2-51.8)	0.73
Iringa	70.6 (62.1-77.9)	24.1 (16.9-33.0)	49.1 (42.9-55.4)	32.3 (28.1-36.5)	0.66
Mbozi	63.3 (49.7-75.0)	21.6 (14.8-30.5)	43.7 (33.4-54.1)	16.9 (8.7-25.0)	0.39
Kahama	72.5 (60.0-82.3)	18.8 (13.2-26.0)	44.1 (34.5-53.7)	22.8 (15.6-30.1)	0.52
Geita	77.3 (66.5-85.4)	16.0 (12.2-20.8)	45.6 (40.7-50.5)	33.6 (28.4-38.8)	0.74
Musoma	80.8 (74.1-86.0)	20.0 (13.8-27.9)	48.6 (42.9-54.4)	39.0 (32.0-46.0)	0.80
**Overall**	**74.5 (71.0-77.7)**	**23.8 (21.2-26.7)**	**49.2 (46.3-52.0)**	**32.8 (29.9-35.8)**	**0.66**

[1-5] Descriptions of mosquito net indicators are listed on Table 1
